# Relationship to physical and psychological pain as factors of deviant behavior in Russian female adolescents

**DOI:** 10.1192/j.eurpsy.2022.695

**Published:** 2022-09-01

**Authors:** E. Rasskazova, V. Sadovnichaja

**Affiliations:** 1 Mental Health Research Center, Medical Psychology, Moscow, Russian Federation; 2 Moscow State University, Clinical Psychology, Moscow, Russian Federation

**Keywords:** relationship to pain, female adolescents, deviant behavior, psychological factors

## Abstract

**Introduction:**

Borderline personality manifests in female adolescence and youth by higher frequency of deviant behaviors and suicidal ideations. Psychological models suggests that both perception and relationship to physical pain (Joiner, 2005, O’Connor, Kirtley, 2018, Galynker, 2017) as well as psychological pain (Eisenberger et al., 2003) could increase the risk.

**Objectives:**

This study concentrates on the relationship between relationship to physical and psychological pain and reported deviant behavior in female adolescents.

**Methods:**

204 female adolescents (13-21 years old) filled checklist appraising alcohol use, drug use, aggressive behavior, suicidal ideations and emotional difficulties (Cronbach’s alphas .67-.89), Interpersonal Needs Questionnaire (Van Orden et al., 2012), Discomfort Intolerance Scale (Schmidt et al., 2006), The Pain Catastrophizing Scale (Sullivan et al., 1995).

**Results:**

Elder females more frequently reported substance use (r=.23-.28) and less frequently aggressive behavior (r=-.19) while suicidal ideations were unrelated to age. Females reporting higher perceived burdensomeness and emotional difficulties also reported higher alcohol use (r=.25-.29), aggressive behavior (r=.37-.42) and suicidal ideations (r=.64-.84). Thwarted belongingness correlated with suicidal ideations (r=.50) and aggressive behavior (r=.26). Higher alcohol use was associated with catastrophizing of pain in the form of magnification and helplessness (r=.17) while suicidal ideations and aggressive behavior were related to ruminations, magnification and helplessness (r=.23-.33). Only correlations between aggression and pain catastrophizing remained significant after statistical control of psychological pain (r=.15-.22).

**Conclusions:**

After control for psychological pain, only aggressive behavior is related to catastrophizing of physical pain. Study is supported by Russian science Foundation, project 22-28-01524.

**Disclosure:**

Study is supported by Russian Science Foundation, project 22-28-01524.

